# Is Cenobamate the Breakthrough We Have Been Wishing for?

**DOI:** 10.3390/ijms22179339

**Published:** 2021-08-28

**Authors:** Nicola Specchio, Nicola Pietrafusa, Federico Vigevano

**Affiliations:** 1 Rare and Complex Epilepsy Unit, Department of Neuroscience, Bambino Gesù Children’s Hospital, IRCCS, Full Member of European Reference Network EpiCARE, 00165 Rome, Italy; nicola1.pietrafusa@opbg.net; 2 Department of Neuroscience, Bambino Gesù Children’s Hospital, IRCCS, Full Member of European Reference Network EpiCARE, 00165 Rome, Italy; federico.vigevano@opbg.net

**Keywords:** cenobamate, drug-resistant epilepsy, anti-seizure medications (ASMs), clinical trials, epilepsy

## Abstract

Close to one-third of patients with epilepsies are refractory to current anti-seizure medications; however, trials with cenobamate suggest effectiveness in such patients with focal onset seizures. We searched for data published or otherwise reported on cenobamate and outlined these here. Despite being marketed in the USA, few studies are yet published in full, and trials are ongoing. Nevertheless, cenobamate showed potential for a high degree of efficacy in reducing seizures with an unprecedented seizure-free rate of up to 28%. Rare cases of hypersensitivity reactions seen in early trials seem to be avoided by the current recommended titration schedule. Other adverse events were rated mild-to-moderate and most commonly included dizziness, drowsiness, and headache. If data are confirmed in further published trials, cenobamate will be a welcome new treatment and further analyses may identify those that will benefit the most.

## 1. Introduction

Recent advances in the development of aetiology-specific drugs for epilepsy substantiate the strategy of targeted drug discovery. Nevertheless, the monogenic epilepsies targeted are uncommon, so the demand for new anti-seizure medications (ASMs) for the most common polygenic and non-genetic (acquired) epilepsies remains unfulfilled. Many patients (>30%) are refractory to current ASMs despite more than 20 s-generation ASMs having been introduced into clinical practice over the last three decades, including 10 new ASMs between 2008 and 2020 [1]. Patients with epilepsy are at increased risk of morbidity, mortality, and a decreased quality of life. They are also more likely to have cognitive deficits, psychiatric disorders, and emotional and psychosocial difficulties [2]. 

Cenobamate is a new ASM recently approved by the US Food and Drug Administration (FDA; November 2019) for the treatment of focal onset seizures in adults, and is currently being reviewed by the European Medicines Agency (EMA) as adjunctive treatment for this indication [3]. Two controlled studies supported a significant reduction in focal seizures with cenobamate in patients with epilepsy who continued their previously prescribed ASMs [4,5].

This narrative review outlines available cenobamate study results from preclinical animal models through to phase 3 clinical studies. While covering the mechanisms of action, pharmacokinetics, efficacy, and safety of cenobamate, we have focussed on the practical information we think will aid those in their first use of this welcome addition.

**Figure 1:**
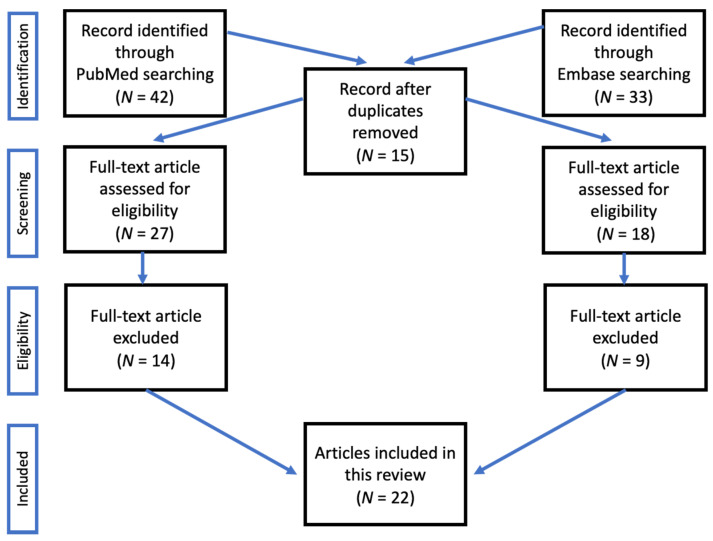
Flow diagram of study selection process.

The PubMed and Embase databases (January 2006 to February 2021) were searched using the following terms: cenobamate, Xcopri (trade name in the USA), and YKP3089 (development code). Article types included in the search output were preclinical studies, clinical trials, observational studies, review articles, and systematic reviews/meta-analyses. Articles were included only if they were published in the English language and evaluated cenobamate with respect to pharmacology, pharmacokinetics, efficacy, and safety in animals or humans. Search results from MEDLINE (via PubMed) and EMBASE^®^ yielded 23 articles (see Figure 1) [4,5,6,7,8,9,10,11,12,13,14,15,16,17,18,19,20,21,22,23,24]. 

## 2. Mechanism of Action

Cenobamate is a novel tetrazole-derived compound with one chiral centre. Dual, complementary mechanisms of action may contribute to its anti-seizure activity [6,10]. The exact mechanism of action is unknown, but cenobamate reduced neuronal excitability in hippocampal CA3 neurons of rats by enhancing the fast and slow inactivation of sodium channels and preferentially inhibiting the persistent component of the sodium channel current [18]. Moreover, cenobamate is a positive modulator of high affinity GABA_A_ receptors from rat hippocampal tissue, acting through allosteric changes from binding at a non-benzodiazepine site [23].

## 3. Experimental Models

Preclinical models of epilepsy suggest anticonvulsant activities of cenobamate to be wide-ranging:

1. Cenobamate prevented:
  - In mouse models, seizures induced by maximal electro-shock (MES), with ED_50_ values of 9.8 mg/kg intraperitoneally;
  - In rat models, seizures induced by MES, with ED_50_ values of 1.9 mg/kg per os [24].
2. In mice, cenobamate prevented clonic seizures induced by the subcutaneous administration of pentylenetetrazole (PTZ) and picrotoxin, with ED_50_ values of 28.5 and 34.5 mg/kg i.p., respectively. In rats, clonic seizures induced by PTZ and status epilepticus induced by lithium/pilocarpine were both abated by cenobamate, with ED_50_ values of 14 and 7 mg/kg i.p., respectively [24].
3. Cenobamate was effective in two models of focal seizures [24]. The ED_50_ value in the hippocampal kindled rat model was 16.4 mg/kg i.p. In the mouse psychomotor seizure model, the ED_50_ value at 6 Hz 22 mA was 11 mg/kg i.p. An increase in the current to 32 or 44 mA in this mouse model had little influence on the potency of cenobamate (ED_50_ values = 18 and 17 mg/kg i.p., respectively).
4. In the genetic absence epilepsy rat from Strasbourg (GAERS) model, the number and cumulative duration of spike-and-wave discharges characteristic of absence seizures were reduced with cenobamate dose-dependently [7,25].

## 4. Pharmacokinetics 

Cenobamate is highly soluble in aqueous solutions. Oral cenobamate is almost completely absorbed (88%) from the gut and is distributed widely with low intra- and inter-subject variability [8,14,26]. An increase in single doses of cenobamate ranging from 5 to 750 mg increased non-linearly the area under the curve (AUC) for the plasma concentration of cenobamate over time. About 2 weeks of once-daily dosing was required for steady-state plasma concentrations, after which both AUC and peak plasma concentration (C_max_) increased proportionally with the therapeutic dose range (100–400 mg/d). Median time to peak concentration (t_max_) is between 1 and 4 h. The volume of distribution from oral administration is around 40–50 L, with 60% binding to plasma protein, which is independent of plasma concentration in vitro [14,20,26].

Cenobamate undergoes extensive metabolization by glucuronidation and by oxidation. Glucuronidation is primarily via uridine 5′-diphospho-glucuronosyltransferase (UGT) UGT2B7 (plus a minor contribution from UGT2B4). Oxidation is via cytochrome P450 (CYP) enzymes CYP2E1, CYP2A6, and CYP2B (minor contributors: CYP2C19 and CYP3A4/5). However, following oral administration of radiolabelled cenobamate, unchanged cenobamate still accounted for >98% of the total AUC of radioactivity in plasma [14,20,26].

The elimination of cenobamate is largely in urine (87.8%) as metabolites, with only 6.8% of the dose being eliminated in urine as unchanged cenobamate. The terminal half-life of cenobamate is 50–60 h, indicating feasibility for once-daily dosing [8,14,20,26].

The pharmacokinetics of cenobamate were unaffected to any degree likely to be clinically relevant with respect to age (based on data from individuals aged 18–77 y), sex, race/ethnicity, weight/body surface area, or following a high-fat meal. Hepatic impairment increased exposure in mildly and moderately impaired subjects by 1.9- to 2.3-fold, respectively. Renal impairment increased exposure in mild and moderately impaired subjects by 1.4- to 1.5-fold [20,26].

**Figure 2:**
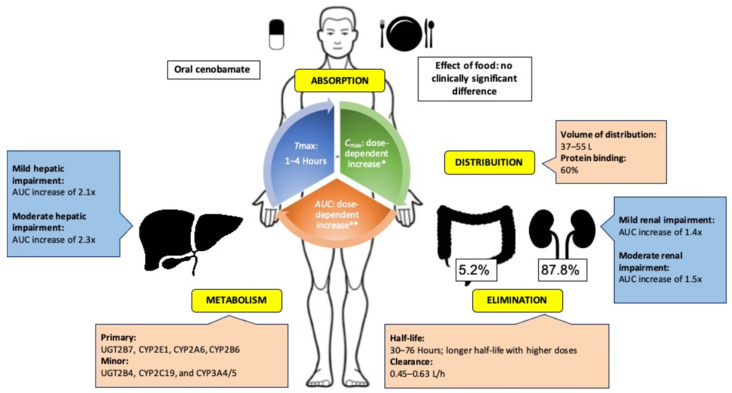
Pharmacokinetics of cenobamate.

Figure 2 shows a summary of absorption, distribution, metabolism, and elimination findings for cenobamate.

## 5. Drug Interactions

Cenobamate may be an inducer of CYP2B6 and CYP3A4 and an inhibitor of CYP2C19, according to a CYP probe study [26]. Importantly, these non-clinical study findings have not yet been proven clinically.

Cenobamate decreased the AUC of the CYP2B6 substrate bupropion by 39% (C_max_ reduced by 23%) and the AUC of the CYP3A4 substrate midazolam by 72% (C_max_ reduced by 61%) [26]. Consequently, increased doses may be required for these and other known substrates of CYP3A4 and CYP2B6 after the initiation of treatment with cenobamate. Importantly, CYP3A4 induction means that alternatives to oral contraceptives should be recommended to women, since a reduction in the AUC of oestrogens and progestins by more than 50% has been associated with CYP3A4 [27]. Increased doses of lamotrigine (LTG) will probably be required with cenobamate, since population pharmacokinetics predict a 21% to 52% decrease in LTG levels [26]. An unconfirmed report of drug interactions with oral contraceptives and LTG suggests that cenobamate also induces UGT metabolism. Levetiracetam levels are also projected to decrease by 4% to 13%, though this is probably not clinically meaningful [26].

Similarly, cenobamate increased the AUC of the CYP2C19 substrate omeprazole by 107% (C_max_ increased by 83%) [26]. Hence, dose reductions of CYP2C19 substrates may be required with cenobamate, a notable example being the active metabolite of clobazam (N-desmethyl clobazam) [28]. In contrast, the co-administration of warfarin (a CYP2C9 substrate) and cenobamate did not affect the pharmacokinetics of either drug [26]. 

Potential drug interaction between cenobamate and selected ASMs was investigated within five phase 1 open-label studies that included 80 healthy volunteers [20]. Valproic acid and cenobamate showed no significant pharmacokinetic interaction. Carbamazepine (CBZ) exposure was reduced by 24% when co-administered with cenobamate, but the plasma AUC of cenobamate was not significantly affected by CBZ. Phenobarbital (PB) AUC was increased by 37% with cenobamate, and PB reduced cenobamate AUC by 15%. Similarly, phenytoin (PHT) exposure was increased by 84% by cenobamate, and PHT reduced cenobamate by 28% [16]. In summary, co-administration with cenobamate will likely require doses of CBZ to be increased and doses of PHT or PB to be decreased (see Table 1) [16,20].

Dose-dependent QT-interval shortening occurred with cenobamate; 31% of healthy volunteers had a QT-interval shortening of 20 ms or more with 200 mg once daily, whereas this was increased to 66% of healthy volunteers taking 500 mg once daily [26]. No healthy volunteer had reductions in the QT interval below 300 ms. Nevertheless, caution is prudent with concomitant QT-shortening drugs for which additive effect or effect modification remain an unknown possibility [26]. 

## 6. Efficacy Data

### 6.1. First Clinical Evidence

A proof-of-concept single-blinded phase IIa study (clinicaltrials.gov identifier NCT00616148) with white male or female adult patients with photosensitive epilepsy (mainly generalised) demonstrated the efficacy of single doses of cenobamate (250 or 400 mg) to suppress photosensitivity either fully or partially in either all (250 mg) or half (400 mg) of four patients tested per dose, whereas 100 mg resulted only in partial suppression within one of three patients tested [15]. 

### 6.2. Randomised Double-Blind Trials

Two phase II, multicentre, randomised, double-blind, placebo-controlled parallel-group trials evaluated the seizure-frequency reduction potential of adjunctive cenobamate given once daily in adults with uncontrolled focal-onset seizures. In other words, the placebo group continued usual standard of care that remained unadjusted throughout the study and the studies were on additional effects to patients classified as uncontrolled.

#### 6.2.1. Phase 2 Study by Chung et al.

This phase 2 study (C013; NCT01397968) compared daily dosing with a maintenance dose of cenobamate 200 mg/d with daily dosing with placebo in 221 adult patients across 40 centres in the USA, India, Republic of Korea, and Poland [5]. Adjunctive treatment duration was 12 weeks, which comprised a 6-week dose-titration period and a 6-week period at maintenance dose.

Eligible patients had epilepsy for a minimum of two years and specifically diagnosed treatment-resistant focal epilepsy—three or more focal seizures per month without 21 consecutive days of seizure freedom—and stable-dose treatment with one to three ASMs (continued through the study). Treatment-related exclusions were current use of PHT or PB, use of felbamate (unless initiation of continuous use exceeded 18 months prior to the study), vigabatrin use within the past year, and continuous use of felbamate for <18 months, and repeated intermittent use of benzodiazepines as a rescue treatment within the month prior to the patient’s first visit. Other exclusion criteria included status epilepticus in the past year, a history of substance abuse, and a high risk of suicide.

The baseline frequency of focal seizures was established during an 8-week baseline period. Patients randomised to the cenobamate group were initiated on cenobamate 50 mg/d (higher than the currently recommended starting dose) and planned to increase by 50 mg every two weeks during the titration period to achieve the target dose of 200 mg/d maintained for the next 6 weeks. Importantly, titration to 200 mg/d was not compulsory and about a third (37%) of patients did not reach this dose but continued the study on lower doses (discontinuation was similarly <10% in both groups), which hinders comparison across studies.

##### Relative Change in Seizure Frequency

**Figure 3:**
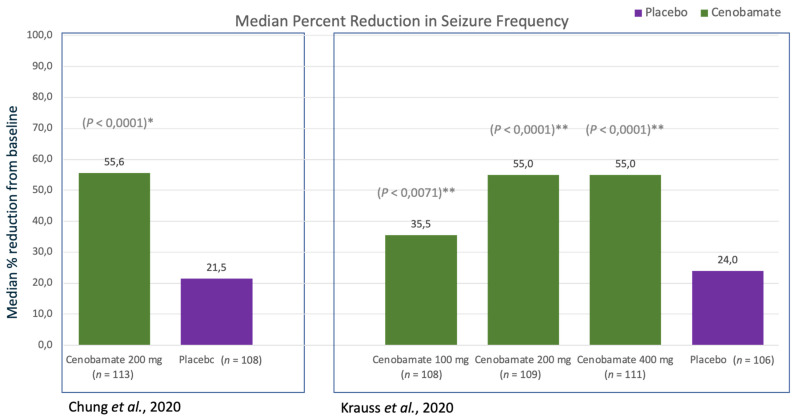
Efficacy data showed as median percentage reduction in seizure frequency from randomised, controlled, double-blind trials [4,5]. * 12-Week treatment period (6-week titration + 6-week maintenance phase), ** 18-Week treatment period (6-week titration + 12-week maintenance phase).

The primary outcome was the median percentage reduction from baseline in 28-day seizure frequency in the intention-to-treat (ITT) population over the 12-week treatment period. Patients in the cenobamate 200-mg/d group (66.7% achieved this dose) had a statistically significant reduction in seizure frequency per 28 days from baseline compared with placebo (−55.6% vs. −21.5%; *p* < 0.0001). In absolute terms, the cenobamate group (*n* = 113) reduced median seizure frequency per 28 days from 7.5 to 3.8 (absolute reduction of 3.7) compared with the placebo group’s (*n* = 108) reduction from 5.5 to 5.0 (absolute reduction of 0.5), which suggests an attributable absolute reduction in median seizure frequency per 28 days of 3.2. Any measure of variation was not reported, though baseline seizure frequency ranged widely from 0 to 187 for the cenobamate group and from 2 to 237 for the placebo group. Notably, the absolute difference between median baseline values was itself 2.0, which may suggest that randomisation failed with respect to exchangeability between the two groups for this measure (see Figure 3).

One secondary outcome was the median normalised percentage seizure reduction by specific seizure type. Patients in the cenobamate group had a significantly greater reduction from baseline compared with placebo in secondary generalised tonic–clonic seizures (77% vs. 33% (*n* = 37–38); *p* = 0.0117), focal unaware seizures (56% vs. 21% (*n* = 87–89); *p* = 0.0009), and focal aware seizures (76% vs. 28% (*n* = 26–30); *p* = 0.0448).

##### Responder Rate

**Figure 4:**
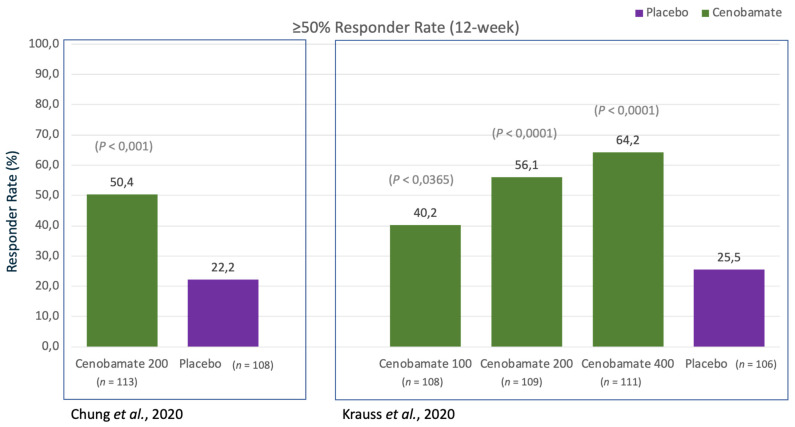
Efficacy data showed as ≥50% responder rate from randomised, controlled, double-blind trials [4,5].

Another secondary outcome was the responder rate (classified as a ≥50% reduction in seizure frequency) during the 12-week treatment period. Patients in the cenobamate group had a ≥50% responder rate of 50.4% compared with 22.2% in the placebo group (odds ratio (OR) 3.94, 95% confidence interval (CI) 2.14–7.24, *p* < 0.0001). During the 6-week maintenance period, post hoc analysis showed that seizure-free (100% response) rates were higher in the cenobamate group than the placebo group (28.3% vs. 8.8%; OR 5.35, 95% CI 2.27–12.64, *p* < 0.0001). Other post hoc analyses included ≥75% and ≥90% responder rates during the 6-week maintenance period, which also suggested positive OR in favour of cenobamate. Analyses with the completer (per protocol) population were concordant with those with the ITT population (see Figure 4).

#### 6.2.2. Phase 2 Study by Krauss et al.

The second phase 2 study (C017; NCT01866111) was a dose–effect study of adjuvant treatment with cenobamate at maintenance doses of 100, 200, and 400 mg/d compared with placebo in approximately 100 adult patients/group across 107 centres in 16 counties across five continents (Asia, North America, Europe, and Australia) [4]. Adjunctive treatment duration was 18 weeks, which comprised a 6-week dose-titration period and a 12-week period at maintenance dose (twice as long as the previous study), and data were analysed over both the 18-week and 12-week periods.

Recruitment criteria were similar to the previous study, with the adjusted exclusion of status epilepticus if within the previous 3 months and additional exclusion of a history of psychogenic or non-epileptic seizures, the presence or history of Lennox–Gastaut syndrome, a central nervous system disease deemed to be progressive that may confound study results, and previous exposure to cenobamate. To avoid possible drug interactions, patients were additionally excluded if they had taken PB or PHT within the month prior to screening. 

Prior to randomisation, as in the first study, seizure frequency and seizure type were evaluated over 8 weeks at baseline. Patients (*n* = 437) were randomised (1:1:1:1) to cenobamate 100, 200, or 400 mg/d or placebo. Initially, per protocol, cenobamate was started at 100 mg/d and the dose increased by 100 mg each week to the target maintenance dose; however, following a blinded review of the first nine patients, the protocol was amended to an initial dose of 50 mg/d with dose increases of 50 mg each week until 200 mg/d and then increases of 100 mg each week in patients randomised to 400 mg/d. This was to reduce the risk of potential drug reaction with eosinophilia and systemic symptoms (DRESS). As before, titration to the assigned dose was not compulsory and patients could continue the study on lower doses; however, only the 400 mg/d group had a median modal dose (i.e., average most frequent dose) lower than nominally categorised with 300 mg (IQR 150–400 mg) over 18 weeks or 400 mg (IQR 200–400 mg) over the 12-week maintenance phase.

Baseline characteristics were comparable between groups. Of 437 patients randomised, 74% were taking either two or three concomitant ASMs. The most common ASMs were levetiracetam (LEV) (43%), lamotrigine (LTG) (32%), and carbamazepine (CBZ) (28%).

This study included two different primary outcome analyses (modified ITT) according to the requirements of regulatory authorities: standardised relative change over the 18-week treatment period (for FDA) and responder (50%) rate over the 12-week maintenance-phase period (for EMA). In total, 434 patients were included in the modified ITT population (18-week period) and 397 patients were included in the modified ITT maintenance-phase population (12-week period).

##### Relative Change in Seizure Frequency

One primary outcome was the median percentage change in focal seizure frequency from baseline per 28 days during the 18-week double-blind treatment. Compared with placebo (median –24%, IQR −45 to −7%), the median reduction from baseline for each cenobamate group was statistically strongly evidenced to be larger (−36% (IQR −63 to −15%) for 100 mg/d, *p* = 0.0071; −55% (−73 to −23%) for 200 mg/d, *p* < 0.0001; −55% (−85 to −28%) for 400 mg/d, *p* < 0.0001). In absolute terms, these suggest reductions of 3.4 (from 9.5 to 6.1), 6.1 (from 11.0 to 4.9), and 5.0 (from 9.0 to 4.0) in median seizure frequency per 28 days for cenobamate 100 mg/d, 200 mg/d, and 400 mg/d, respectively.

A secondary outcome analysis looked at the percentage change solely over the 12-week maintenance phase (following the titration), which the investigators suggest better represents the potential efficacy of the 400 mg/d group. Compared with placebo (median −27%, IQR −51 to −8%), the median reduction from baseline for cenobamate 100 mg/d was statistically weakly evidenced to be larger (−42% (IQR −65 to −11%), *p* = 0.0537), but remained statistically strongly evidenced to be larger for the higher doses (−57% (−78 to −25%) for 200 mg/d, *p* < 0.0001; −63% (−97 to −28%) for 400 mg/d, *p* < 0.0001). In absolute terms, these are reductions of 4.0, 6.3, and 5.7 in median seizure frequency per 28 days for cenobamate 100 mg/d, 200 mg/d, and 400 mg/d, respectively.

These results taken together with those of the previous study suggest that cenobamate 200 mg/d would appear to be the most favourable of these doses for efficacy. In this second study, baseline median values across groups ranged from 8.4 (placebo) to 11.0 (cenobamate 200 mg/d)—a difference of 2.6—however, this dose-ranging study shows a dose effect independent of the baseline values, which suggests that potential effect modification due to higher baseline values seems unlikely to have influenced this inference even if randomisation had again failed to make the groups exchangeable for this measure (see Figure 3).

##### Responder Rate

The other primary outcome was the responder rate (defined as the percentage of patients achieving ≥50% reduction from baseline in focal seizures) during the 12-week maintenance phase. The placebo group had 25% responders, whereas 40%, 56%, and 64% were responders in the cenobamate 100-, 200-, and 400-mg groups, respectively. Compared with the placebo group, the OR for being a responder in the cenobamate groups was 1.97 with 100 mg/d (95% CI 1.08–3.56, *p* = 0.0365), 3.74 with 200 mg/d (95% CI 2.06–6.80, *p* < 0.0001), and 5.24 with 400 mg/d (95% CI 2.84–9.67, *p* < 0.0001). The responder rate over the 18-week period was a secondary outcome and results were similar to the 12-week maintenance phase. The dose effect is clear to discern and the values for 200 mg/d are concordant with those in the first study, despite the difference in trial period and actual dose variability.

As one additional pre-specified outcome, seizure-free (100% response) rates were achieved by only one patient (1%) in the placebo group compared with four patients in the 100-mg group (4%; *p* = 0.3688), 11 patients in the 200-mg group (11%; *p* = 0.0022), and 20 patients in the 400-mg group (21%; *p* < 0.0001). Similar dose–effect trends were shown with the pre-specified outcome of ≥75% and ≥90% response rates (see Figure 4).

### 6.3. Ongoing Open-Label Efficacy Studies

Both phase II studies had the option for patients to enrol in open-label extensions for both efficacy and safety analyses.

#### 6.3.1. Extension of First Phase II Study (C013; NCT01397968)

From those completing the first phase II study (201 patients), 149 patients continued in the open-label extension with an intended dose of cenobamate of 200 mg/d. Of these patients, 76 were naive to cenobamate, whereas the remaining 73 had cenobamate previously. The number of patients still being studied was 86 as of April 2018, with a median of 61 months of cenobamate treatment and a median dose of 200 mg/d. Interim long-term safety outcomes have been reported in brief, but efficacy data are yet to be reported [29].

#### 6.3.2. Extension of Second Phase II Study (C017; NCT01866111)

Long-term efficacy and safety outcomes have been reported in brief for 355 patients who entered the open-label extension of the second phase II trial. Of these patients, 90 were naive to cenobamate, whereas the majority (265 patients) had cenobamate previously. The number of patients still being studied was 230 as of April 2018. Reasons for discontinuing treatment (*n* = 125) were lack of efficacy in 15.5% and adverse effects in 6.8%. 

After six months in the open-label extension, patients transitioning from placebo had a median seizure reduction of 63%, which compares well with the 65.7% reduction observed in patients continuing cenobamate first administered in the original study and provides strong evidence for the efficacy of cenobamate. At 25 to 30 months, the median seizure frequency among all enrolled patients was a reduction of 76%, suggesting that seizure reduction is not only sustained over time but potentially improves. Comparable to the outcome reported within the original study for the 400-mg/d group, 20.2% of patients in the extension had achieved seizure freedom at 25 to 30 months [29].

## 7. Tolerability and Adverse Effect Profile

The safety profile of cenobamate is seemingly predictable and generally well tolerated. The most frequent treatment-emergent adverse events (TEAEs) reported during the two phase II trials (C013, C017) and their extensions were mild-to-moderate in intensity, CNS-related, and were dose-dependent. Combining both trials, TEAEs occurring in 10% or more of patients were dizziness, somnolence, headache, fatigue, and diplopia. In descending dose order, among the most common TEAEs in the double-blind phase of the dose-range study (C017), somnolence occurred in 37%, 21%, and 19% of patients (8% in the placebo group), whereas dizziness was reported in 33%, 20%, and 18% of patients (14% in the placebo group) [4]. Neither trial had any clinically meaningful changes from baseline in haematology, clinical chemistry, or laboratory values [4,5].

Open-label extensions of the trials C013 and C017 are ongoing (see above), though adverse events (AEs) after 2–5 years in these remained primarily neurological and occurred in 88–89% of patients. Discontinuation as a result of AEs in the extensions occurred in 6.8% and 9.4% of patients, respectively [10]. The most frequent TEAEs (≥10%) common to both open-label extensions (respectively) were dizziness (32.2%, 32.7%), headache (26.2%, 11.8%), somnolence (20.8%, 23.9%), and fatigue (10.7%, 14.9%); additionally for C013 were viral upper respiratory tract infection (17.4%), upper respiratory tract infection (13.4%), nausea (10.7%), and urinary tract infection (10.1%); and additionally for C017 were diplopia (13.8%) and gait disturbances (11%) [10].

During early clinical development, three confirmed cases of DRESS (two healthy volunteers and one patient with epilepsy) occurred among the first 953 adults exposed. Cases of DRESS were associated with cenobamate being initiated at a high starting dose and/or titrated weekly or faster [9]. With the two cases in healthy volunteers, one had a starting dose of cenobamate 200 mg/d that was increased every 5–7 days by 100 mg/d, whereas the other started at 50 mg/d and increased weekly by 50 mg/d. The patient with epilepsy began with cenobamate 100 mg/d and increased weekly by 100 mg/d.

### Ongoing Phase III Open-Label Safety Studies

**Figure 5:**
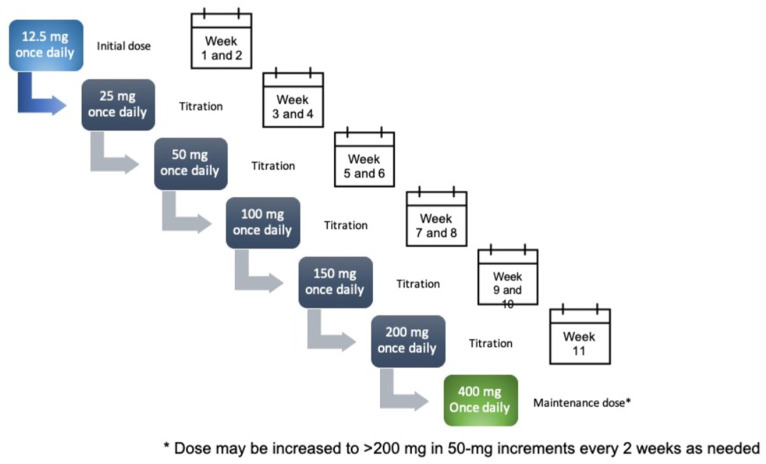
FDA-approved cenobamate dosing schedule for focal onset seizures.

A more gradual titration schedule was developed in hope of reducing the incidence of DRESS and is now the currently recommended dosing titration schedule (Figure 5) [26]. This schedule was investigated in an ongoing phase III, multicentre, open-label safety trial at 139 centres across five continents (C021; NCT02535091) [9]. Similar to the previous studies, this study enrolled adult epilepsy patients with uncontrolled focal epilepsy currently taking 1–3 ASMs. Inclusion limitations were also similar to the previous studies with the major exception that the study initially enrolled only patients taking PHT and PB before later enrolling those with other ASMs. 

Interim safety results have been published [9]. All hypersensitivity reactions were reviewed monthly for DRESS but no cases were identified. Hence, lowering both the starting dose and the titration rate of cenobamate seems to have mitigated the risk of DRESS. As of April 2018, 1339 patients received at least one dose of cenobamate, 82.9% of patients (*n* = 1110) were exposed to cenobamate for ≥6 months, and 80% of patients (*n* = 1078) were still continuing in the study. The most frequent TEAEs (incidence ≥10%) were somnolence (28.1%), dizziness (23.6%), fatigue (16.6%), and headache (11.4%).

Secondary outcomes from this ongoing study include effects of the titration of cenobamate on the pharmacokinetics of PHT and PB. Although 43% and 30% of patients had their doses of PHT or PB, respectively, reduced (following a protocol restriction of 25–33% reduction), mean plasma PHT/PB levels were generally comparable before and after the cenobamate titration period, suggesting stable doses are achievable with relatively simple modifications [9].

## 8. Conclusions and Expert Opinion

Adjunctive treatment with cenobamate shows potential for a high degree of efficacy in reducing seizures when treating patients with refractory focal epilepsy—reductions to a degree greater than has been observed with other ASMs [11,13,17,19,30,31,32]. However, this requires confirmation from head-to-head trials. The interpretation of short-term adjunctive trials is challenging and sensitivity analyses for differences in baseline seizure frequency would have been beneficial in these trials. Nevertheless, reductions in monthly seizure frequency of up to 55% and seizure-free rates of up to 28% [7,21] in patients with refractory epilepsy despite the concomitant use of 1–3 ASMs deserve our attention. High responders appeared to benefit with high QOLIE scores [12]. Cenobamate seems to have a safety profile comparable to other ASMs. Lowering the starting dose of cenobamate and slowing the titration rate (see Figure 5) seem to have managed the risk of previously seen cases of DRESS, for which no additional cases have occurred to date with long-term treatment. Vigilance during dose titration is nevertheless still required. Assuming real-world use reflects observations from these clinical studies, cenobamate is likely to become a stalwart medication within the next five years.

Interestingly, in the first phase II study (C013), rates of seizure freedom were 9.1% for placebo and 27.5% for the cenobamate group [5], and seizure freedom increased with dose in the second phase II study (C017) [4]. The open-label extension of study C017 suggests sustained seizure reduction frequency and seizure freedom (20.2%) to 25 to 30 months [29]. To the best of our knowledge, achieving seizure freedom in approximately one in every five patients is higher than observed in any previous placebo-controlled trial of ASMs (albeit methods differ). For example, the highest incidence of seizure freedom was 6.5% in recent systematic reviews and meta-analyses of placebo-controlled trials for focal seizures [30,31,32].

Seizure frequency could be halved by cenobamate within the first 4 weeks of titration if a post hoc analysis from study C017 proves representative [4]. This not only suggests that even low doses of cenobamate can be beneficial, but may underlie an early predictor of whether patients will respond, thereby potentially avoiding needless titration to higher doses in eventual non-responders. This observation warrants further investigation.

## 9. Future

The publication of data from the ongoing trials yet to report is anticipated. A phase 3 trial is currently recruiting to investigate potential benefits of cenobamate for generalised epilepsy [33]. Analyses of patient subgroups may identify those who benefit the most from this new addition, which may well represent a breakthrough that we have been long awaiting.

## Figures and Tables

**Table 1:** Pharmacokinetic drug interaction of cenobamate.

Drug/Substrate	Effect of Cenomabate on Drug/Substrate	Effect of Drug/Substrate on Cenomabate	Expected Effects	Clinical Recommendation
**Lamotrigine**	↓ plasma concentrations	-	↓ concentrations by 21–52%	Monitoring and ↑ the dosage of lamotrigine
**Carbamazepine**	↓ plasma concentrations	-	↓ AUC of 24%	Monitoring and ↑ the dosage of carbamazepine
**Phenytoin**	↑ plasma concentrations	↓ cenobamate concentrations	▪ PHT ↑ AUC of 84% and Cmax of 70%. ▪ AUC of cenobamate ↓ by 28%	Monitoring and gradually ↓ PHT dosage by up to 50% during titration
**Phenobarbital**	↑ plasma concentrations	↓ cenobamate concentrations	▪ PB ↑ AUC of 37% and Cmax of 34%. ▪ AUC of cenobamate ↓ by 15%	Monitoring and ↓ dosage of phenobarbital
**Desmethylclobazam**	↑ plasma concentrations	-	↑ concentrations of N-CLB	Monitoring and ↓ dosage of clobazam
**CYP2B6 Substrates**	↓ plasma concentrations	-	↓ AUC of 39% and Cmax of 23%	↑ the dosage of CYP2B6 substrates
**CYP3A Substrates**	↓ plasma concentrations	-	↓ AUC of 72% and Cmax of 61%	↑ the dosage of CYP2B6 substrates
**CYP2C19 substrates**	↑ plasma concentrations	-	↓ AUC of 107% and Cmax of 83%	↓ dosage of CYP2C19 substrates
**Oral Contraceptives**	↓ plasma concentrations	-	↓ concentrations of oral contraceptives are expected because of CYP3A4 induction	Women should use additional or alternative non-hormonal birth control
**Drugs That Shorten the QT Interval**	Additive effect on QT interval shortening	-	Cenobamate has demonstrated dose-dependent shortening of the QT interval	Caution should be used when administering cenobamate and other drugs that shorten the QT interval
**CNS Depressants**	Additive effect of CNS depressants	-	Cenobamate has demonstrated dose-dependent adverse effects that primarily affect the CNS	CNS depressants should be used cautiously when used in combination with cenobamate

CYP: Cytochrome P, CNS: central nervous system, AUC: area under the curve, PHT: phenytoin, PB: phenobarbital, N-CLB: norclobazam, ↓: reduction, ↑: increase.

## Data Availability

Not applicable.
